# The Association between Intrahousehold Food Allocation Social Norms and Thinness among Young Adolescent Girls: A Community-Based Study

**DOI:** 10.4314/ejhs.v33i6.4

**Published:** 2023-11

**Authors:** Hanna Gulema, Meaza Demissie, Alemayehu Worku, Tesfaye Assebe Yadeta, Nebiyou Fasil, Yemane Berhane

**Affiliations:** 1 Department of Global Health and Health Policy, Addis Continental Institute of Public Health, Addis Ababa, Ethiopia; 2 School of Public Health, Addis Ababa University, Addis Ababa, Ethiopia; 3 School of Public Health, Haramaya University College of Health and Medical Sciences, Harar, Ethiopia; 4 Department of Epidemiology and Biostatistics, Addis Continental Institute of Public Health, Addis Ababa, Ethiopia

**Keywords:** Intrahousehold food allocation, mothers/caregivers, social norms, young adolescent girls, thinness

## Abstract

**Background:**

Inequalities in food allocation related to social norms among household members significantly affect the nutritional status and well-being of the vulnerable members of the household, such as adolescent girls. This study assesses the association between social norms related to intrahousehold food allocation and young adolescent girls' thinness.

**Materials and Methods:**

The study involved 1,083 pairs of mothers/caregivers and young adolescent girls. Data were collected using a structured and pretested questionnaire. Multilevel mixed-effect logistic regression analysis was employed to examine associations using STATA/SE V.14 statistical software. In addition, a stratified analysis was done to investigate the effect of social norms on thinness in food-secure and food-insecure households.

**Result:**

The overall prevalence of young adolescent girls' thinness was 15.70% (95% CI 13.52–17.86%). Young adolescent girls' thinness was associated with mothers'/caregivers' conformity to inequitable intrahousehold food allocation social norms in food-secure households [Adjusted odds ratio (AOR): 1.43, 95% CI: 1.14–1.80] but not in food-insecure households.

**Conclusion:**

Nearly 16% of adolescent girls were thin. Mothers/female caregivers conforming to inequitable intrahousehold food allocation social norms compromise the nutritional status of adolescent girls, particularly in food-secure households. The lack of statistically significant association in food-insecure households hints at the need to address inequality in food-secure households while addressing food shortage to improve the nutritional status of adolescents in low-income countries. We also recommend more studies in different sociocultural contexts to fully gather the evidence for adopting appropriate policies and practices.

## Introduction

Undernutrition among young adolescent girls is a significant public health concern, particularly in low-income countries (1). Thinness during adolescence can affect linear growth and make adolescents more likely to develop health complications in their future lives. Yet, despite the negative consequences of social norms on nutritional status in female adolescents, it has received less attention in low-income countries (2).

According to the WHO Global Health Observatory data, the prevalence of thinness among adolescent girls (10-19 years) varies across countries and regions. Among female adolescent girls globally (WHO), the prevalence of thinness is 8.1%. South-East Asia has the highest prevalence of thinness (17.1%), and the Americas has the lowest prevalence (1.2%); the prevalence in Africa was 4.7% (3). In Ethiopia, a study reported 24.9% thinness among adolescent girls (4).

The factors related to adolescent thinness include consumption of poor quality and quantity foods, discriminatory intrahousehold allocation, gender-based norms, place of residence, lower socioeconomic status, household food insecurity, and inequitable social norms (5,6). Household-level social norms influence individual members' food allocation, which impacts health and nutritional well-being (7).

Social norms are informal rules that influence behavior in a given community and relate to perceptions of what most people do (descriptive norms) and expectations of what people should do (injunctive norms) (8). Household food allocation is shown to be affected by prevailing social norms (9). Food allocation social norms in low-income settings generally favor male household members to eat more frequently and larger portions. In comparison, females eat less portions and less frequently and eat last in the household (10). As a result, young adolescent girls from low-income countries experience monotonous or less diversified diets dominated by few available staples (11). The Ethiopian National Food Consumption Survey and other studies in the country reported that a monotonous diet is typical among Ethiopian households (12). A monotonous diet may reflect community cultural eating habits and lower availability of various foods during the survey period (13). Inter-household food allocation social norms are enforced by reference groups or norm holders, including mothers/caregivers (14). Therefore, poor nutrition associated with inequitable social norms will likely have intergenerational effects (15).

Emerging evidence suggests that the nutritional status of young adolescent girls can be improved by implementing interventions that modify social norms. In societies where social norms are significant drivers of behaviors and decision-making, understanding the association between caregivers' food allocation norms and adolescent thinness is critical (16,17). However, little documented evidence exists about the link between caregivers' food allocation social norms and adolescent girls' thinness. Therefore, this study aims to assess the association between social norms related to intra-household food allocation and thinness among young adolescent girls in rural Ethiopia.

## Materials and Methods

**Study setting, design, and population**: The data for this analysis were drawn from a large quasi-experimental research implemented in four districts in West Hararghe, Oromia regional state, Ethiopia (18). The West Hararghe communities are mainly dependent on agriculture. They produce maize and sorghum for subsistence and khat and coffee as cash crops. The area is often affected by drought and is chronically food insecure. The population predominantly follows Islam and has a patriarchal structure. It is not uncommon for adolescents to get married at around the age of 15 years. The study used data from the baseline cross-sectional study of the parent study. This study involved only young unmarried adolescent girls aged 13 and 14 and their corresponding mothers/ female caregivers. The eligibility criteria for the study include being a permanent resident for at least six months and being an unmarried adolescent girl (age 13-14 years).

**Sample size and sampling procedure**: The sample for this study was calculated using a double population proportion formula with the following conservative assumptions: the proportion of exposure (inequitable social norms) among controls (not thin) to be 50%, 95% level of confidence, 80% Power, case-to-control ratio of 1:2, and minimum detectable odds ratio of 1.5. The calculated sample size was 918 (306 cases and 612 controls). Accordingly, the available data from the baseline survey was considered sufficient to conduct this study.

The study population for the baseline survey was selected in a multistage cluster sampling procedure. In the first stage, 114 clusters (localities, locally referred to as local development zones) were selected using simple random sampling after obtaining a complete list of the clusters in the selected districts from the respective district health office. In the second stage, a complete household listing was done in each selected cluster to identify households with eligible adolescent girls. Then, 30 households with eligible adolescent girls were randomly selected from the prepared list using a computer-generated random number. If there were more than one eligible adolescent girl in the household, one was selected randomly using the lottery method.

**Data collection instrument and data collection procedure**: Data were collected using a structured and pretested questionnaire designed to collect information on adolescent girls, caregivers, and households. The tools were first developed in English and then translated into the regional language, Afaan *Oromo*. An open data kit (ODK) electronic data collection software was used to capture the data during the survey. The adolescent girl's questionnaire gathered information on sociodemographic variables such as age, educational status, and adolescent anthropometry. The household-level questionnaire collected data on household food insecurity and wealth and was administered to the head of the household. Female caregivers' information was gathered on sociodemographic and household food distribution social norms. Data were collected by enumerators fluent in the written and spoken *Afaan Oromo* language. Anthropometric measurements were taken using UNICEF standard scales. Training was provided to the data collectors prior to the fieldwork. Standardization was done for the data collectors responsible for anthropometry measurements. Weight and height measurements were taken by a pair of data collectors. Additionally, two rounds of measures were taken for each adolescent girl to ensure the accuracy of the anthropometric data. The pretest was conducted in a similar setting that was not included in the study. The study team carried out supervision throughout the data collection period. A field manual was prepared to guide the data collection in the field.

**Measurements**: Household food allocation social norms were measured using a Likert scale categorized into five coded groups: strongly disagree as “4”, disagree as “3”, agree as “2”, and strongly agree as “1”, and do not know as “0”. Those who refused to answer this question were treated as missing responses. Eleven items were included for measuring descriptive social norms and 10 for measuring injunctive social norms related to household food distribution. Nine items were included to measure mothers'/caregivers' attitudes towards intrahousehold food allocation norms. A summary of all items was used to create a descriptive norm score ranging from 0 to 44, an injunctive norm score ranging from 0 to 40, and mothers'/caregivers' attitude score ranging from 0 to 36. Therefore, the higher value of the composite norms and attitude scores indicate unfavorable household food distribution social norms and negative attitudes towards equitable food allocation (19). The descriptive food distribution norm scale showed an acceptable reliability measure of Guttmann's Lambda 2 of 0.79. The injunctive food distribution norm scale showed an acceptable reliability measure of Guttmann's Lambda 2 of 0.84. The measure of female caregivers' attitudes towards intrahousehold food allocation for adolescent girls was found to be reliable, with a Guttmann's Lambda 2 of 0.77. Thinness was defined as having a BMI-for-age z-score below -2 standard deviation (SD) from the median of the WHO Growth Reference for school-aged children and adolescents. Anthropometric measurements analysis were conducted on adolescent girls using WHO AnthroPlus (20).

Household food insecurity was measured using the Household Food Insecurity Assessment Scale (HFIAS) developed by Food and Nutrition Technical Assistance (FANTA) for use in developing country contexts (21).The household food insecurity assessment scale categorizes households into four levels: food-secure, and mild, moderately, and severely food-insecure.

**Statistical analysis**: Data analysis was done using STATA/SE version 14 statistical software. Sampling weight was produced because of the complex survey nature of the data. Intra-cluster correlation (ICC) was checked to see variation between clusters. Chi-square was used to see whether there is a significant difference in adolescents' thinness proportion between food-secure and food-insecure households. The association between female caregivers' household food distribution social norms and adolescent girls' thinness was examined by controlling for variables such as adolescent-related factors (adolescent girl's age, educational status), female caregivers' factors (female caregivers' education, marital status, religion, income, and age) and household factors (wealth index, and household food insecurity). Multilevel mixed effect logistic regression was applied to examine the association between female caregivers' household food allocation norms and adolescent girls' thinness because of the clustered nature of the data. Variables with a p-value <0.05 in a multivariable analysis were considered statistically significant, and the adjusted odds ratio (AOR) with a 95% Confidence Interval (CI) was estimated to measure the strength of the association. The mixed effect logistic regression model assumptions, such as outliers and multicollinearity, were checked and fulfilled. Outliers were assessed on a graphical plot, and the variance inflation factor (VIF) was computed to check multicollinearity. The VIF value was less than 10, which was tolerable.

**Ethics statement**: The research protocol was approved by the Ethical Review Board of the Addis Continental Institute of Public Health (Ref No. ACIPH/IRB/005/2016). Permission for data use was sought and secured from the main study principal investigator. Informed verbal consent was sought from the participants before the commencement of the study. Mothers/caregivers consented to their participation and permitted their adolescent girls' participation. In addition, assent was also obtained from adolescent girls. No personal identifiers were included in the analysis dataset. We adhered to the Helsinki Declaration guidelines while conducting this study.

## Results

**Sociodemographic characteristics**: A total of 1,083 mothers/caregivers and young adolescent girls' pairs were included in the analysis. The average age of adolescents is 13.5 years (±SD 0.5) and 37.9 years (±8.4) for mothers/caregivers. About 13.4% of adolescent girls and 85.0% of mothers/caregivers never attended school. More than 94.3% of the mothers/caregivers were married, and 86.7 were Muslims. In addition, 83.8% of mothers/caregivers had no formal employment (Table 1).

**Table 1:** Socio- demography of adolescent girls and their female caregivers' and household characteristics, West Hararghe, Oromia, Ethiopia

Variables	Number (%)
Adolescent girls Age	
13 years	562 (51.9)
14 years	521(48.1)
Mean age	13.5 SD±0.5
Adolescent Education	
Never Attend	145(13.4)
Primary and above	938(86.6)
Female caregivers' educational status	
Never Attend	921(85.0)
Primary and above	162(15.0)
Female caregivers' Marital status	
Never married	62(5.7)
Ever married	1,021(94.3)
Female caregivers' religion	
Christian	144(13.3)
Muslim	939(86.7)
Female caregivers' Occupation	
Farmer or family farm work	89(8.2)
Wage work	86(7.9)
No formal employment	908(83.8)
Wealth index in tercile	
Lowest	363(33.5)
Middle	363(33.5)
Highest	357(34.0)

**Figure 1:**
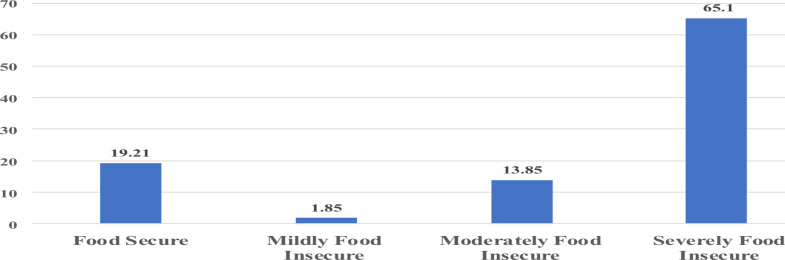
Proportion of household food security level, Hararghe, Oromia, Ethiopia

**Household decision-making and food security participants status**: Regarding household decision-making, 63.86% of the mothers/caregivers reported higher self or joint decision-making. In 83.21% of the households, male family members were reported to eat first (Table 2). Only 19.21% of the households that participated in the study were food secure, while 65.10% were severely food insecure (Figure 1).

**Table 2:** Household Decision-Making and Food Security Status, West Hararghe, Oromia, Ethiopia

Variables	Number (%)
Decision on household income	
Myself and jointly	689 (63.7)
Husband	390 (36.1)
Who eat first in the household	
Adolescent, adult women and jointly	181 (16.8)
Male family members	897 (83.2)
Household food security	
Food Secured	208 (19.2)
Mildly Food Insecure	20 (1.6)
Moderately Food Insecure	150 (13.6)
Severely Food Insecure	705 (65.1)

**Intrahousehold food allocation social norms**: The intrahousehold food allocation injunctive norms mean score was 22.48(SD± 5.55), from the maximum score of 44. Overall, 45.80% of the mothers/caregivers scored above the mean, indicating that they believe other mothers/caregivers expect them to conform with inequitable intrahousehold food allocation norms. Whereas the intrahousehold food allocation descriptive norms mean score was 25.73 (SD±5.41), from the maximum score of 40. Overall, 48.38% of the mothers/caregivers scored above the mean, indicating that they believe that most other mothers/caregivers indeed conform with inequitable intrahousehold food allocation norms. Mothers'/caregivers' attitude mean score towards intrahousehold food allocation norms was 16.19 (SD±4.23). Overall, 45.89 % of the mothers/caregivers hold a negative attitude towards equitable intrahousehold food allocation, implying no differentiation between female and male adolescents (Table 3).

**Table 3:** Maximum score, mean with standard deviation and proportion of Female caregivers harmful intrahousehold food allocation social norms and attitude, West Hararghe, Oromia, Ethiopia

Variables	Maximum score	Mean (±SD)	Proportion % (CI)
Female caregivers harmful intrahousehold food allocation social norms (Injunctive)	44	22.48(±5.55)	
Proportion of study participants who scored above the mean for injuctive norms			45.80(42.83,48.77)
Female caregivers harmful intrahousehold food allocation social norms (descriptive)	40	25.73(±5.41)	
Proportion of study participants who scored above the mean for descriptive norm			48.38(45.40,51.36)
Female caregivers' attitude towards intrahousehold food allocation	36	16.19(±4.23)	
Proportion of Female caregivers' who scored above the mean for attitude towards equitable intrahousehold food allocation			45.89(42.92,48.86)

**Figure 2:**
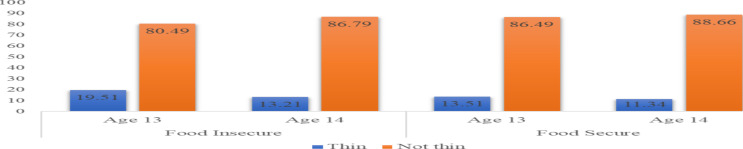
Proportion of adolescent girls' thinness by their age and household food security level, Hararghe, Oromia, Ethiopia

Household food allocation norms associated with adolescent girls' thinness: The overall prevalence of young adolescent girls' thinness was 15.70% (95% CI 13.52–17.86%). More than 80% of the households were food insecure (80.79%). In stratified analysis, the prevalence of thinness among adolescent girls was slightly higher in food-insecure households (16.46%) than in food-secured households (12.50%), though the difference was not statistically significant. The age-stratified result shows that adolescent girls' thinness was greater among girls aged 13 years in both food-secure and food-insecure households. However, only the proportion of thinness between 13 and 14 years of adolescents in food-insecure households was statistically significant (P-value = 0.012) (Figure 2).

The overall (pooled) association between intrahousehold food allocation norms and adolescent thinness was not statistically significant (AOR 1.06; 95% CI: (0.99,1.14). Similarly, the association was not statistically significant in food-insecure households (AOR 1.03, 95% CI: 0.95-1.11). However, the association was statistically significant in the food-secure households (AOR 1.43, 95% CI: 1.14–1.80). The unstratified and stratified models controlled for household wealth, adolescents' age and education, female caregivers'/mothers' education, religion, occupation, the decision on household income, attitude towards intrahousehold food allocation, marital status, and cluster effect (Table 4).

**Table 4:** Pooled and stratified Bivariable and Multivariable Analysis of female caregiver intrahousehold food allocation norms and adolescent thinness in West Hararghe zone Oromia, Ethiopia

Categories	Crude Odds Ratio (95% Confidence Interval)	*Adjusted Odds Ratio (95% CI)
**Pooled**		
Mothers/caregivers' harmful intrahousehold food allocation norm	0.99 (0.95,1.03)	1.06(0.99,1.14)
**Food secured households**		
Mothers/caregivers' harmful intrahousehold food allocation norm	1.05(0.94,1.17)	1.43(1.14, 1.80)
**Food insecure household**		
Mothers/caregivers' harmful intrahousehold food allocation norm	0.98(0.94,1.02)	1.03 (0.95, 1.11)

* The model adjusted for adolescent age, adolescent education level, mothers/caregivers' education, marital status, religion, and occupation. Households' characteristics such as Female caregivers' decision on household income, Female caregivers' attitude towards intrahousehold food allocation, female caregivers' descriptive food allocation norm, who eat first in the household, and Wealth tercile

## Discussion

This study showed the prevalence of thinness among adolescent girls to be 15.70% (95% CI 13.52%–17.86%). We also found that conforming to inequitable food allocation social norms was associated with young adolescent girls' thinness in food-secure households. However, the association was not statistically significant in food-insecure households.

The observed prevalence of thinness in female adolescent girls is consistent with the pooled prevalence (17.7%; 95% CI: 14.6, 20.8) reported in a recent systematic review (22). However, other studies reported lower and higher prevalence rate; for instance, 7.1% in Dangla, northern Ethiopia (23), 10.3% in Debark, Northwest Ethiopia (24),15.8% in Northeast Ethiopia (25), and 29.2% in Jimma, southwest Ethiopia (26). The variation could be attributed to differences in the food security status of the areas, prevailing social norms, and seasonal variations (27).

Gender-based household food allocation social norms that prioritize male family members are not uncommon in low-income settings (15,28). This preference in patriarchal society emerges from the dominant male's decision-making power, income-generating role, and the perception that males exert more energy in their daily activity than females (29). Thus, the thinness observed in this study may not be unique to the study setting though considerable variations are observed depending on the context, even in the same country.

The observed association in food-secure households indicates that discriminatory food allocation practices have an effect when sufficient food is available to make a meaningful discrimination in the diet of household members. Adolescent girls in households conforming to inequitable food allocation norms would be more vulnerable to malnutrition due to insufficient food to meet their energy and nutritional demands (30). A diet that lacks essential nutrients ultimately leads to thinning of adolescent girls (12,31). In low-income countries, inequitable intrahousehold food allocation social norms are affected by many factors, including scarcity, as explained by the family stress theory (32), or by discriminatory norms, as explained by Festinger's social comparison theory (33). The former explains the lack of association in food insecure households, and the latter explains the observed association in food-secure households.

According to sociological and psychological perspectives and Festinger's social comparison theory, social interaction and observational learning contribute to forming and sustaining social norms (33,34). Conforming to social norms related to intrahousehold food allocation often involves the prospect of discrimination. In turn, discriminatory allocation could happen where food security is better. Therefore, caregivers make decisions regarding discriminatory food allocation among household members in accordance with these social norms (35,36).

The findings also indicate that regardless of the food security status of the households, adolescent girls remain vulnerable to malnutrition, in this case, thinness. Marginal improvements in food security status may exacerbate inequitable intrahousehold food allocation in low-income settings. Furthermore, it is crucial to recognize that these inequitable intrahousehold food allocation norms are deeply rooted, and changing them may require long-term and multi-faceted interventions integrated into sustained poverty alleviation programs.

In this study, the lack of association between inequitable food allocation social norms and adolescent thinness in food-insecure households could also be attributed to inadequate sample size. Also, when food insecurity is severe, the discriminatory capacity of the social norms would be minimal. Everybody gets much less than the recommended diet (37). According to the literature, adolescents with low bargaining power have less control over intrahousehold food allocation and, as a result, are more likely to have poor nutritional status. Conversely, adolescent girls who are more involved in food preparation and cooking are more likely to eat a healthy diet and have better nutritional status. Cultural norms can also be related to taste preferences and unhealthy eating patterns (38). However, this study did not assess adolescent bargaining power, personal preference, and engagement in food preparation.

The observed thinness and its association with social norms could have long-term implications for the health and well-being of adolescent girls and, later to their offsprings. Inadequate nutrition could impact physical growth, micronutrient deficiencies, and compromised immune functions (39). Further, it can negatively impact adolescent girls' future reproductive health and the health of their future children (40).

This study's primary strength was using a quantitative approach to examine the association of interest, which was uncommon in previous studies. However, the study's main limitation is that the social norms measurement was done at one point in time and thus may be influenced by recall bias and social desirability bias. The social desirability bias can potentially obscure the study's ability to detect inequitable intrahousehold food allocation norms between the comparison groups. The effect of the recall bias is difficult to determine; it can go either way.

The bias related to the outcome (thinness) is minimal as data collectors were given intensive training and were standardized; in addition, anthropometric equipment was calibrated regularly. Another issue worth noting is that the WHO 2007 Growth Reference has an inherent limitation as it does not account for the complex racial/ethnic variation across populations during the adolescents' growth spurt.

In addition, the homogeneity of our study population regarding religion, socioeconomic status, ethnicity, language, and agriculture-based way of living makes observing stronger associations difficult; more significant differences would have been detected if the study community was heterogeneous in their social and cultural norms.

In conclusion, about one in six adolescent girls was thin. Conforming to inequitable intrahousehold food allocation social norms threaten the nutritional status of adolescent girls, particularly in food-secure households. No statistically significant association between social norms and thinness in food-insecure households was observed. Addressing inequality in food-secure households while combating food shortage is critical to improving the nutritional status of adolescents in low-income countries. Further studies in different sociocultural contexts are warranted to make broader policy recommendations.
